# Lymphocytes from chronic lymphocytic leukaemia undergo ABL1-linked amoeboid motility and homotypic interaction as an early adaptive change to *ex vivo* culture

**DOI:** 10.1186/2162-3619-3-7

**Published:** 2014-03-11

**Authors:** Claire V Hutchinson, Shiva Natarajan, Suzanne M Johnson, Julie A Adams, Karen S Rees-Unwin, John Burthem

**Affiliations:** 1 Institute of Cancer Sciences, Haematological Oncology, University of Manchester, Level 5 Research St. Mary’s Hospital, Oxford Road, Manchester M13 9WL, UK; 2 Institute of Cancer Sciences, Manchester Academic Health Science Centre, The Christie NHS Foundation Trust, 550 Wilmslow Rd, Manchester M20 4BX, UK; 3 Clinical Haematology, Central Manchester University Hospitals, Oxford Road, Manchester M13 9WL, UK

**Keywords:** CLL, ABL1, Actin, Cytoskeleton, Motility, Homotypic

## Abstract

**Background:**

Those stimuli that together promote the survival, differentiation and proliferation of the abnormal B-lymphocytes of chronic lymphocytic leukaemia (CLL) are encountered within tissues, where together they form the growth-supporting microenvironment. Different tissue-culture systems promote the survival of the neoplastic lymphocytes from CLL, partly replicating the *in vivo* tissue environment of the disorder. In the present study, we focussed on the initial adaptive changes to the tissue culture environment focussing particularly on migratory behaviour and cellular interactions.

**Methods:**

A high-density CLL culture system was employed to test CLL cell-responses using a range of microscopic techniques and flow cytometric analyses, supported by mathematical measures of cell shape-change and by biochemical techniques. The study focussed on the evaluation of changes to the F-actin cytoskeleton and cell behaviour and on ABL1 signalling processes.

**Results:**

We showed that the earliest functional response by the neoplastic lymphocytes was a rapid shape-change caused through rearrangement of the F-actin cytoskeleton that resulted in amoeboid motility and promoted frequent homotypic interaction between cells. This initial response was functionally distinct from the elongated motility that was induced by chemokine stimulation, and which also characterised heterotypic interactions between CLL lymphocytes and accessory cells at later culture periods. ABL1 is highly expressed in CLL lymphocytes and supports their survival, it is also recognised however to have a major role in the control of the F-actin cytoskeleton. We found that the cytoplasmic fraction of ABL1 became co-localised with F-actin structures of the CLL lymphocytes and that the ABL1 substrate CRKL became phosphorylated during initial shape-change. The ABL-inhibitor imatinib mesylate prevented amoeboid movement and markedly reduced homotypic interactions, causing cells to acquire a globular shape to rearrange F-actin to a microvillus form that closely resembled that of CLL cells isolated directly from circulation.

**Conclusion:**

We suggest that ABL1-induced amoeboid motility and homotypic interaction represent a distinctive early response to the tissue environment by CLL lymphocytes. This response is separate from that induced by chemokine or during heterotypic cell-contact, and may play a role in the initial entry and interactions of CLL lymphocytes in tissues.

## Background

Those stimuli that promote the survival, differentiation and proliferation of the abnormal B-lymphocytes of chronic lymphocytic leukaemia (CLL) are encountered within tissues, where together they form the growth-supporting microenvironment (reviewed in [1]). The soluble and structural elements of the CLL microenvironment include accessory cells [2], chemokines [3] and perhaps antigenic stimuli [4,5], as well as contact with other CLL cells [6], and cases with significant infiltration of tissues have adverse clinical outcome [7]. The effective interaction between CLL lymphocytes and their microenvironment therefore depends on directed migration and appropriate cellular contact [3,8,9].

When cultured *ex vivo* at standard cell density, pure preparations of neoplastic CLL-lymphocytes from CLL patients enter apoptotic cell death within 24 hours [10]. Apoptosis is however prevented by the additional presence of relevant accessory cells [3,11], or by culture of the CLL lymphocytes together at high cell density with blood mononuclear cells [10]. The enhanced cell survival within these cultures is believed to derive, at least in part, from direct interaction between cells [6]. Such interactions resemble the behaviour of normal B-lymphocytes at particular developmental stages *in vivo,* in which migration through tissues involves repeated brief homotypic and heterotypic cell contacts, as well as more sustained intercellular interactions [12]. The processes that control movement, interaction and adhesion for cells of the immune system require the dynamic and coordinated rearrangements of the cell cytoskeleton mediated through specific filamentous actin (F-actin) structures. In this regard, there is an emerging recognition that the Abelson kinase (ABL1) has a central role in the organisation of F-actin structures that promote particular aspects of cell motility and interaction in cells of the immune system [13]. ABL1 is highly expressed in the lymphocytes of CLL, and the inhibitor of ABL-family protein tyrosine kinases imatinib mesylate (imatinib) has been shown to induce the apoptosis of neoplastic CLL B-lymphocytes *in vitro*[14]. In CLL, imatinib targets are largely confined to the ABL kinases [15]. We postulated therefore that imatinib might alter F-actin mediated motility or adhesion of CLL lymphocytes.

Recent reports that a redistribution of CLL lymphocytes between blood and tissues accompanies the therapeutic effect of signal-inhibitor agents as part of their clinical effect [16], have further highlighted the importance of understanding how tissue migration and adhesion in CLL is controlled. In the present paper we describe very specific rearrangements of F-actin that are induced in *ex vivo* when CLL lymphocytes are maintained in high-density culture. We show that these rearrangements mediate frequent, but transient, homotypic interactions between the neoplastic CLL B-lymphocytes, and that the morphological appearances of the CLL lymphocytes in high-density culture resemble those of CLL lymphocytes within tissues, and are consistent with the interactions between cells seen in tissues [17]. The F-actin rearrangements we describe are shown to be functionally distinct from those induced by chemokine, and from those that drive heterotypic interactions between CLL cells and accessory cells. We suggest that ABL-dependent adhesion and migration represents a separate pathway of tissue interaction for CLL lymphocytes that may be important in the initial adaptation to the tissue environment.

## Results

### High cell-density culture of CLL B-lymphocytes promotes early shape-change and the formation of transient homotypic interactions

**Figure 1:**
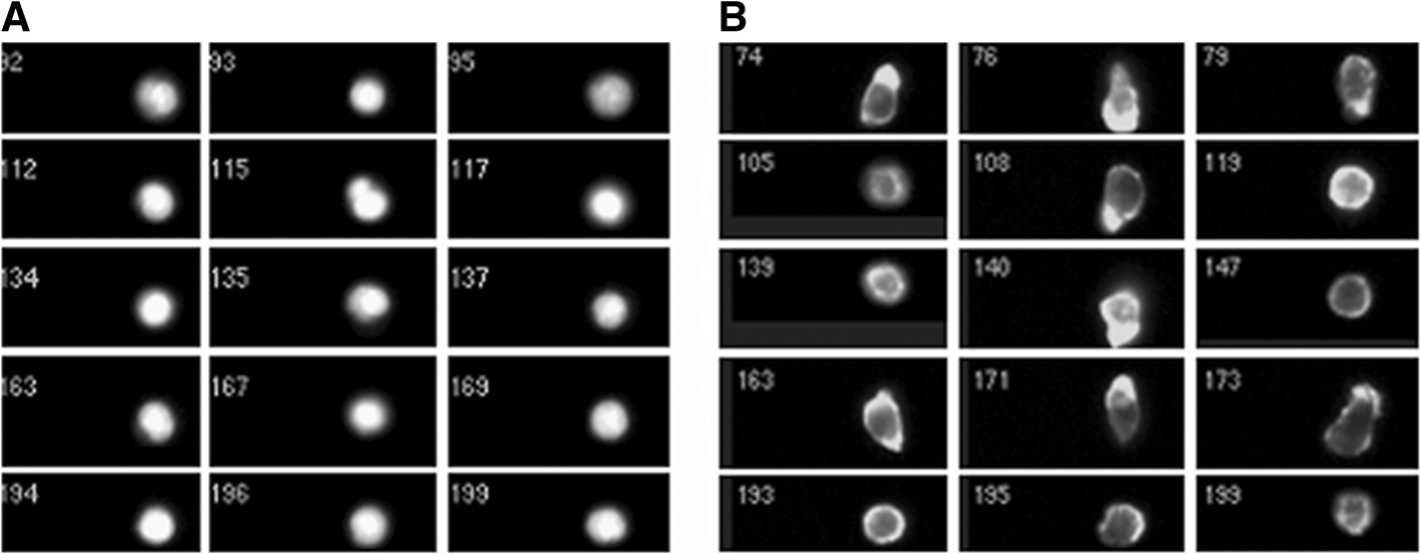
**Changes to cell-shape and cytoskeleton organisation of CLL lymphocytes within high-density culture (fixed cells stained for NBD-phallacidin to show F-actin dependent cell-shape were assessed using imaging flow cytometry:). Panel A**. Images of cells fixed in whole blood directly from the circulation, using a case where >95% of cells were CLL B-lymphocytes. Imaging is restricted to nucleated cells (identified by Dapi fluorescence), and neutrophils and monocytes are excluded by size and shape criteria. The cells demonstrate largely spherical shape and uniform F-actin distribution [confirmed by high aspect ratio: mean 0.84 ± 0.01 where a perfect sphere is 1.0]. **Panel B**. Cell shape from high-density culture: profiles indicate irregular and round cells [overall mean aspect ratio is reduced indicating greater irregularity than circulating cells: 0.69 ± 0.01 (p < 0.00001, n = 560 Mann-Witney U test)]. Cells with polarised F-actin form 46% of cells compared with 4% of cells from circulation (representative of 8 separate experiments using adherent or non-adherent cells).

**Figure 2:**
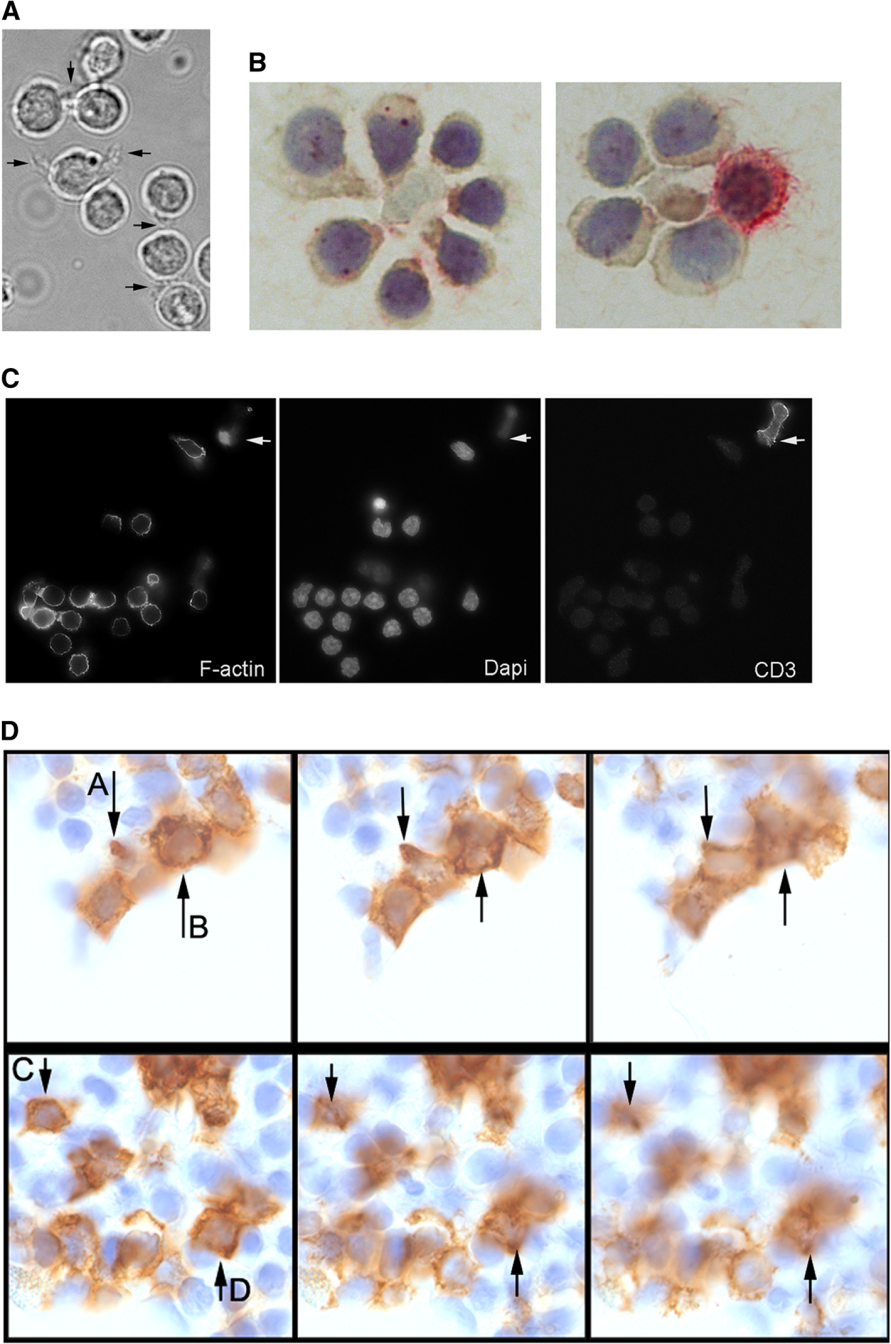
**Homotypic interactions between CLL lymphocytes in culture and within tissues. Panel A**. Phase-contrast image of live cells within culture showing that cytoplasmic projections extend directly between adjacent cells (arrowed). **Panel B**. Immunoperoxidase staining shows that within some small clusters CD4 cells are demonstrated, but these are absent in most cases. **Panel C**. Immunostaining of cell groups demonstrating the homotypic nature of interactions: left panel shows F-actin (Texas Red phalloidin) illustrating irregular cell shape and interactive projections connecting cells; centre panel cell nuclei (dapi); right panel lineage markers - in this example CD3-FITC to show that T lymphocytes are separate from the groups. Groups were demonstrated in all CLL cases tested (n = 8). The majority of groups contain 2–3 cells, but larger groups were also seen. **Panel C**. Lymph node tissue involved by CLL lymphocytes immunostained to demonstrate cell process formation (CD23-peroxidase/DAB – brown, counterstained with hematoxylin). Fine focal depth microscopy shows successive z-planes illustrating contact between adjacent CLL lymphocytes, and irregular cell shape with extended processes. Examples with extended cell processes passing through the tissue planes shown are specifically indicated **(A-D** arrowed on slide).

CLL lymphocytes isolated from blood may successfully be cultured *ex vivo* in the absence of “feeder cell” types, provided cultures employ unselected mononuclear cells and are seeded as high cell-density [10]. In the present study we confirmed those findings, showing that at low cell-seeding densities (2 × 10^5^/ml) fewer than 10% of CLL cells survived after 24 hours; by contrast using high seeding-density (5 × 10^6^/ml) increased survival to a mean of 84 ± 9% (n = 8). Simultaneous morphological observation showed that the cells cultured at high density also underwent a marked shape-change: Cells fixed directly in blood had a globular shape with numerous cell-surface microvilli, within culture however, the cytoskeleton was rearranged to form an irregular shape in which microvilli were replaced by extended cellular projections. These changes were apparent within 2 hours and were seen also in serum-free medium or autologous plasma, but were not observed when cells were seeded at low densities. The shape-changes were confirmed and quantified using mathematical measures. Cells adherent to fibronectin-coated slides were used to reflect aspects of the *in vivo* environment [18,19] (characterised using morphometric measures of fixed cells), or aspirated from live cell culture (characterised using imaging flow cytometry). These measures confirmed that the cell population within culture had a significantly different shape when compared with cells fixed directly in blood (Figure 1A and B). The shape change observed in high-density conditions was accompanied in all cases by the formation of small homotypic cell-groups comprised solely of neoplastic B-lymphocytes in which cells were seen to extend projections in a polarised manner toward adjacent cells (Figure 2A). Interactions between the neoplastic lymphocytes of CLL and CD4 T-lymphocytes are recognised during *in vitro* culture and within tissues *in vivo*[20]. CD4-lymphocytes were relatively rare cells in our cultures and could not alone explain the high frequency interactions observed. Nonetheless, immunostaining did allow these heterotypic interactions to be recognised in our culture system, most frequently seen as part of larger aggregates together with NLCs. In a minority of cases the CD4 cells were seen also to form part of the smaller aggregates (Figure 2B). In most cases however, the small transient groups were comprised solely of neoplastic B-lymphocytes (Figure 2C). In comparable cultures using mononuclear cells from normal individuals, the B-lymphocytes did not show major shape change or group formation. When CLL lymphocytes were examined within *in vivo* sections of bone marrow or lymph node, the neoplastic CLL-lymphocytes also had irregular shape with extended cytoplasmic-projections. These appearances identified homotypic contact between CLL lymphocytes, and resembled those of the CLL cells within high-density culture (Figure 2D).

### The shape-change of cultured CLL lymphocytes reflects dynamic F-actin rearrangement supporting amoeboid motility and interactive filopodia formation

**Figure 3:**
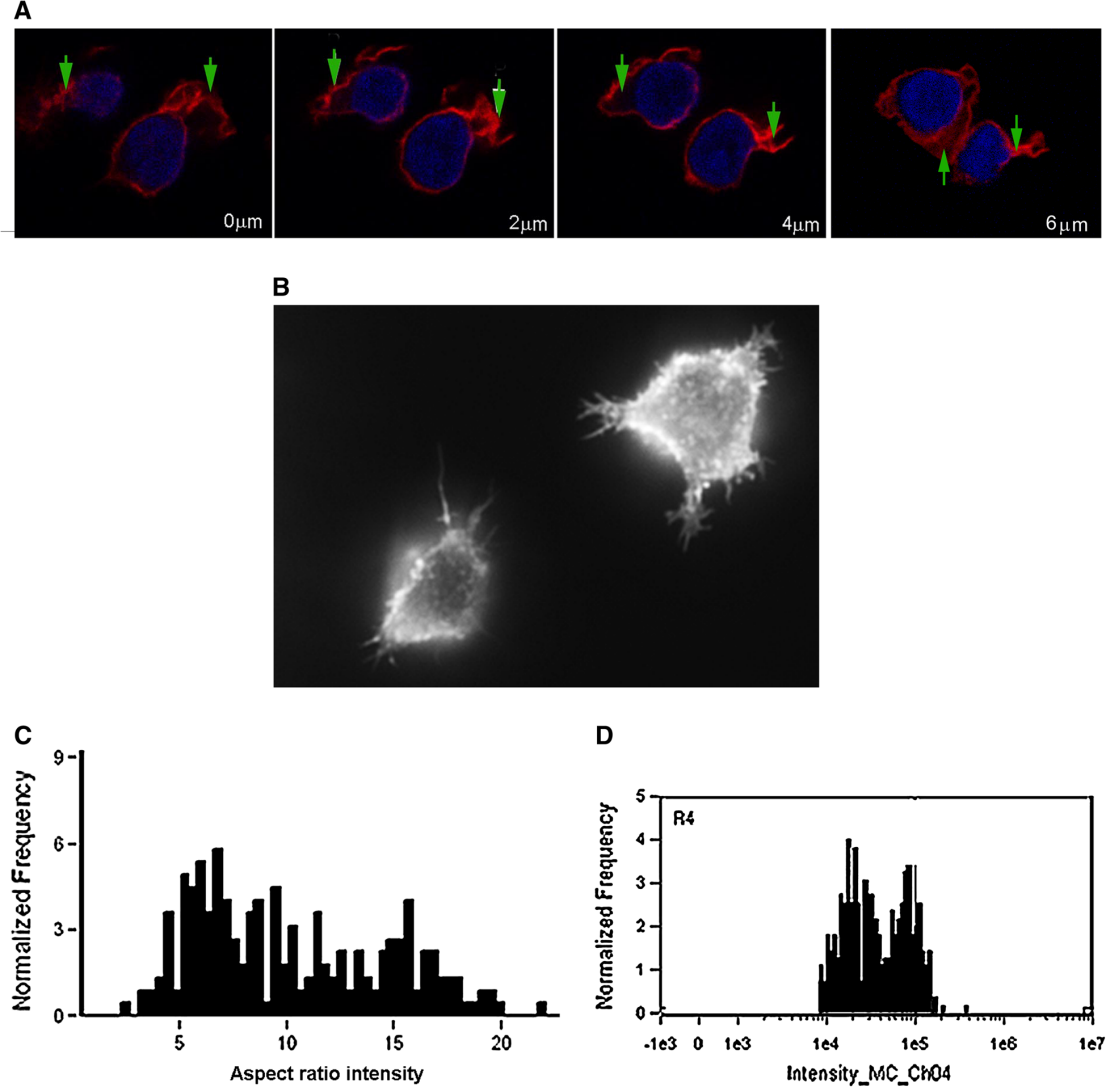
**Detailed analysis of the shape forms and cytoskeletal rearrangement observed for the CLL lymphocytes in culture. Panels A & B.** Confocal microscopy showing F-actin cytoskeletal arrangement of CLL lymphocytes during motility **(A)** and conventional fluorescent microscopy **(B)** (TR-phalloidin probed for F-actin at z planes, Dapi nuclear stain as indicated). F-actin is enriched within extended projections, but no clear lamellipodium or posterior attachment is formed. **Panel C**. Representative histogram of shape change (measured as aspect ratio intensity a weighted measure of shape and F-actin polarisation in which high levels indicate round non-polar cells) revealing that there are two shape-form populations at fixed time points within the culture (confirmed by 4 separate assessments of adherent or aspirated cells from culture). **Panel D**. A similar dual population of F-actin content is indicated by flow cytometric analysis of the cultured cells (Flow cytometric analysis using NBD-phallacidin probe).

**Figure 4:**
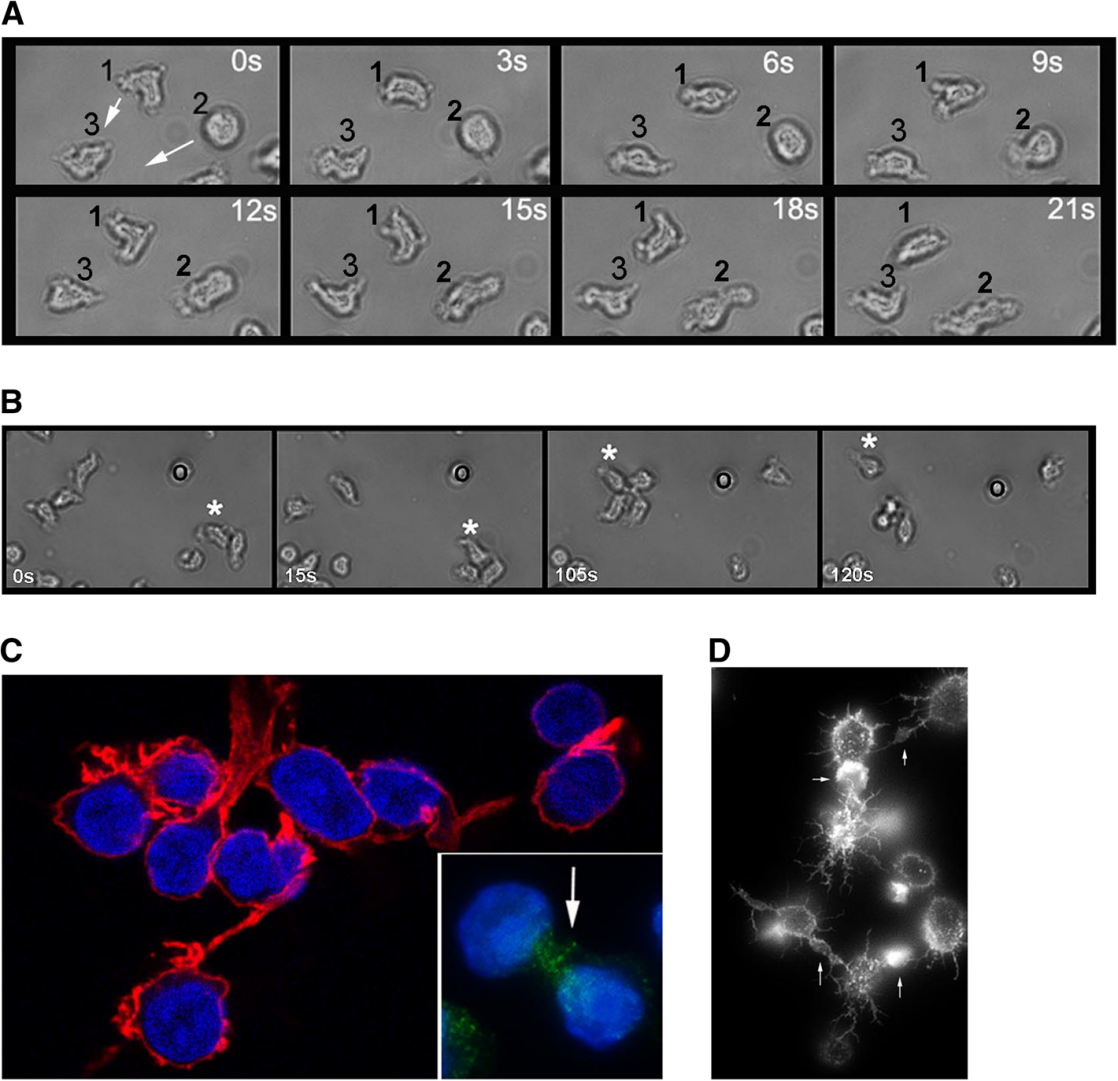
**Analysis of the behavioural significance of the CLL shape change. Panel A**. Time-lapse photomicroscopy of cells within culture illustrating movement of CLL lymphocytes (phase contrast image series). Cells make a transition between round and polar form during typical amoeboid motility. The extension and retraction of processes during motility are clearly seen in cells marked 1 and 2 as they move toward cell 3, note that cell 2 makes a characteristic change from round form to elongated form during the 21 second observation period. **Panel B**. Time-lapse photomicroscopy illustrating transient cell-group formation between CLL lymphocytes (phase contrast image series of 120 second observation). The cell marked with *migrates across consecutively forming groups of 2, 3, 4 cells then back to a single cell. Groups interact by polarised extended projections, and cells show typical transition from elongated to round forms. **Panel C**. Complex filopodial contact during homotypic interactions (confocal microscopy of F-actin, TR-phalloidin probe) Main panel: basal attachment point to the finronectin-coated Plate. F-actin processes (Texas Red phallacidin probe) extend horizontally linking adjacent cells. Inset panel: enrichment for VLA-family integrin receptors CD29-FITC. **Panel D**. Rho kinase inhibition reveals extensive interaction between CLL lymphocytes (TR-phalloidin probe for F-actin). Filopodial retraction is prevented using rho-kinase inhibition (Y27536), a branched network of filopodial connections is revealed (arrows indicate points of intercellular contact where F-actin is specifically enriched).

The functional significance of the shape-change and interactions seen in high-density culture was tested further using cells probed for F-actin then examined using confocal microscopy, or by time-lapse photo-microscopy of live cultured cells. Individual CLL lymphocytes examined by confocal imaging of F-actin showed a characteristic shape change and extension of irregular cytoplasmic projections. F-actin had a partly polar distribution and was specifically enriched within veil-like projections (Figure 3A and B). Analysis of the whole population of CLL lymphocytes in culture however revealed a bi-modal distribution of cell shape and F-actin content, showing that a population of rounded cells with low F-actin content co-existed with irregular shaped cells that had higher F-actin content (Figure 3C and D). Time-interval photo microscopy revealed that these apparently separate round and irregular cell forms in fact formed part of a single population of cells that were in dynamic equilibrium. Over short periods (<20 s) the CLL lymphocytes underwent transition between circular shape and irregular form, undergoing a “random-walk” pattern migration across the tissue culture surface (Figure 4A). Together, these morphological, cytoskeletal and functional findings are typical of the cycles of F-actin polymerisation and relaxation that are seen during amoeboid-type movement. Additional to the changes of amoeboid motility, homotypic group formation was also highly dynamic: Time-lapse observation revealed that the contacts and cell groups were transient, becoming separated after short time periods, following which the cells went on to engage in further homotypic interactions (Figure 4B). The cytoskeletal projections that mediated the homotypic contact were rapidly formed and retracted over periods of seconds, and had the characteristics of typical filopodia, with a narrow central actin core (Figure 4C), and were enriched with integrin adhesion receptors at the point of contact (Figure 4C inset). Extensive interacting networks were seen when retraction of these processes was prevented using the Rho-kinase inhibitor Y-27632 [21] (Figure 4D).

**Figure 5:**
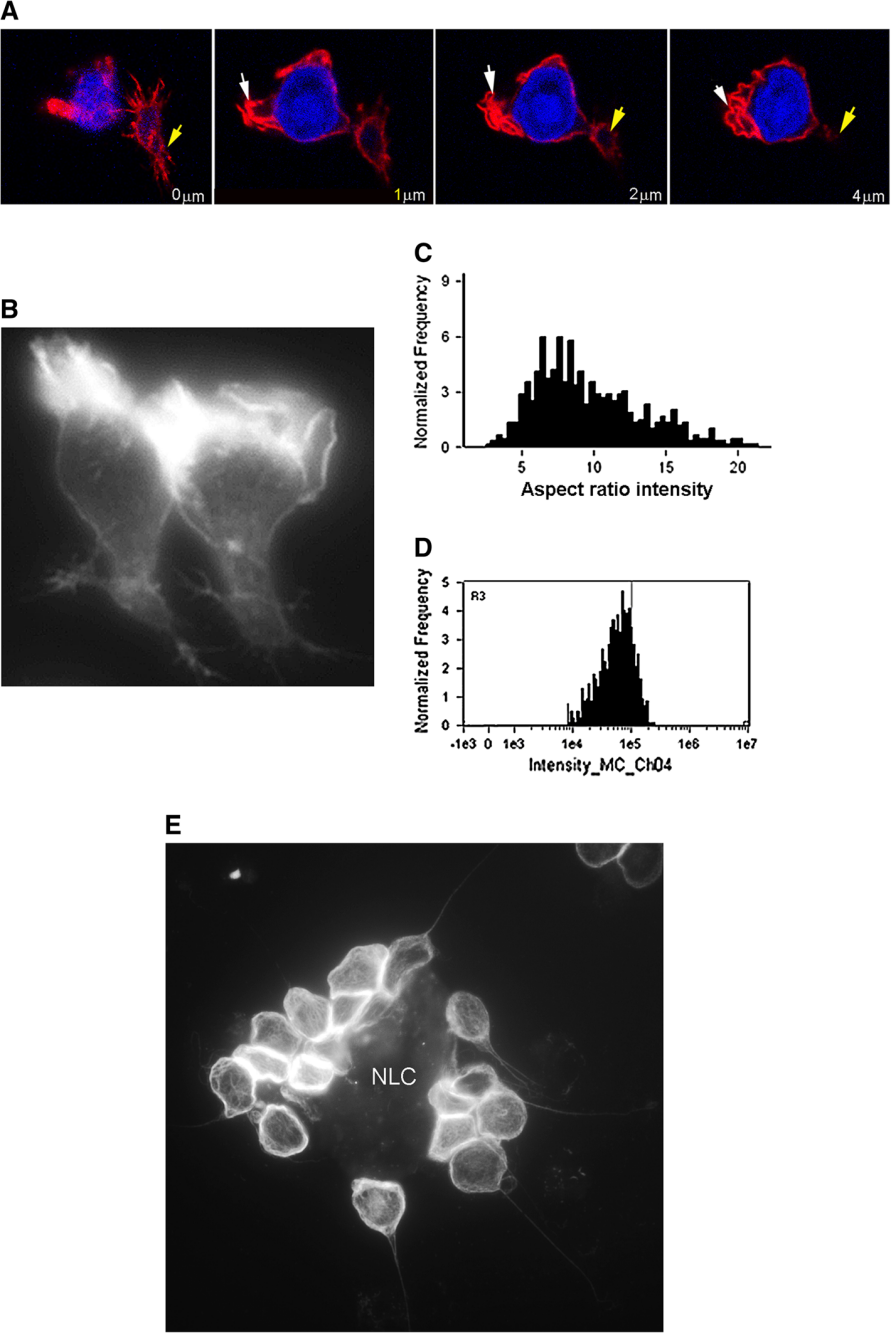
**Cytoskeletal arrangements of CLL lymphocytes treated with chemokine or undergoing heterotypic interaction have specific characteristics that differ from cells undergoing amoeboid motility. Panel A & B** Chemokine treated CLL lymphocytes probed for F-actin (representative confocal microscopic images z planes as indicated, cells probed using TR-phalloidin) **(A)** and conventional immunofluorescence **(B)**. The representative cells were exposed to CXCL12 (30 minutes). In the confocal images F-actin is particularly enriched within an anterior structures forming clear structural folds (white arrows); these may be seen (upper z plane) to form a single structure with linear actin ruffles (lamellaepodium). At the basal plane there is a well-defined posterior attachment point (yellow arrow). Similar changes may also be seen in the conventional fluorescent images. **Panel C**. Cell shape distribution now indicates a single population of elongated cells (aspect ratio intensity measurment as described in Figure 3). **Panel D**. A single distribution of high F-actin intensity is also now present (NBD-phallacidin probe for F-actin assessed by flow cytometry). **Panel E**. CLL lymphocytes interacting with accessory cells in culture have similar elongated and polarised form to chemokine-treated cells. This image is from a later culture point when NLCs are fully formed (7 days). The CLL lymphocytes attached to the central NLC body show clear polarity with anterior interaction and posterior tail, similar to cells treated with chemokine and no filopodia are evident (Texas-Red phalloidin probe for F-actin).

The appearances we observed in early culture were functionally distinct from the chemokine-induced motility that drives the heterotypic interactions between CLL lymphocytes, nurse-like cells (NLCs) and T cells that has been extensively characterised by others [22]. Chemokine-treated CLL B-cells became markedly elongated with clear front-tail polarity, and were characterised by a well-developed anterior lamellipodium and posterior attachment point (Figures 5A and B). Analysis of cell populations confirmed also that they differed in shape and F-actin dynamics from those undergoing amoeboid motility. Cell-shape analysis showed a single distribution peak of cells with a shift to lower aspect ratio when compared with control cells (more elongated appearance), during which the whole population acquired a higher F-actin content in a single distribution peak (Figure 5C and D). These appearances were statistically distinct from the amoeboid shape change we described at earlier time points. Aspect ratio measurement was 0.65 ± 0.01; compared with cells not exposed to CXCL12 (0.69 ± 0.01) (p < 0.01 n = 570). Consistent with the importance of chemokines in the heterotypic interactions between CLL cells and accessory cells, marked front-tail polarity similar to chemokine-treated cells was observed when CLL cells interacted within heterotypic groups (Figure 5E).

### ABL1 supports the amoeboid motility and homotypic interactions of CLL lymphocytes, but is not required for the separate elongated motility directed by chemokines

**Figure 6:**
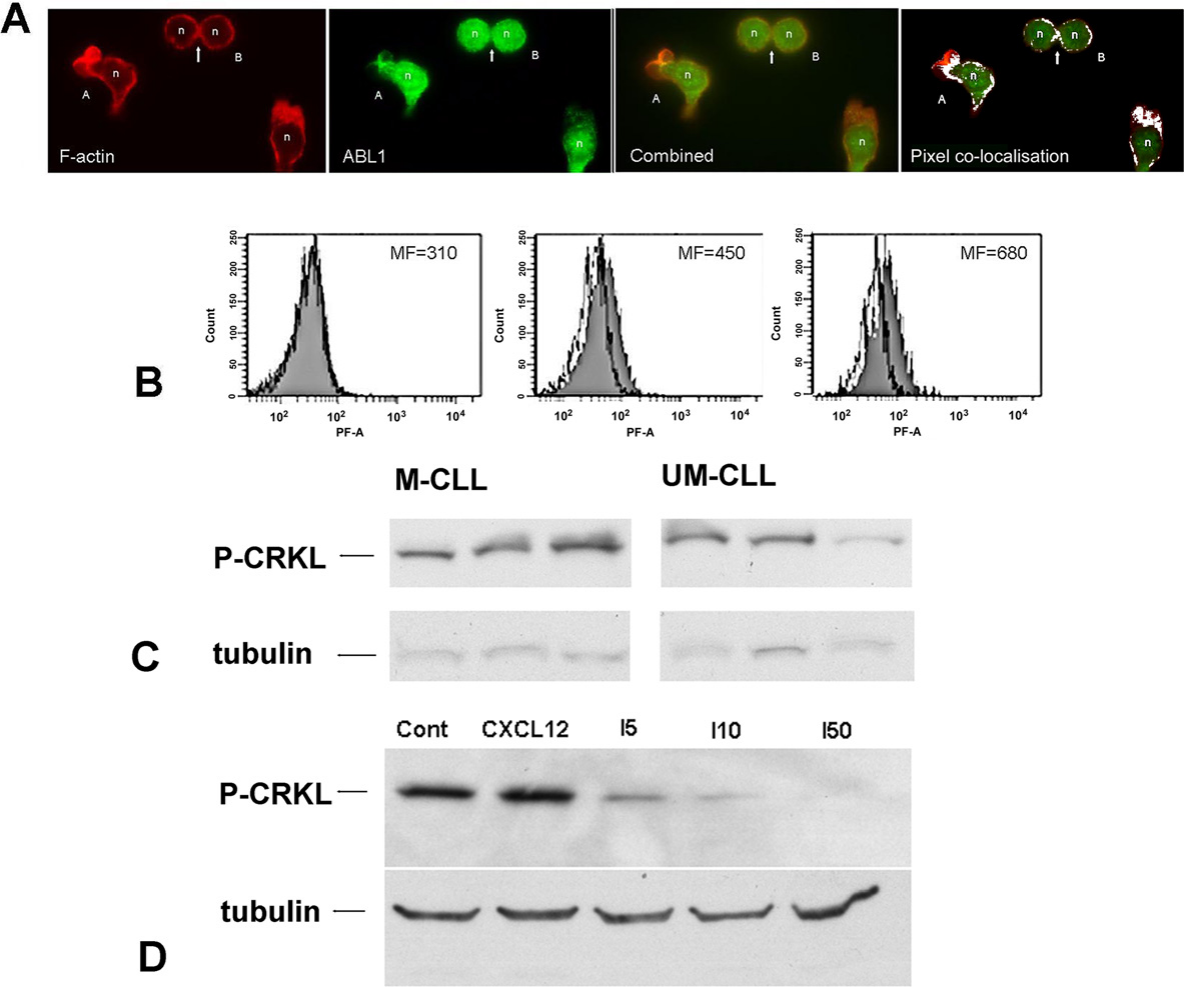
**ABL1 is localized with F-actin structures in CLL cells and the substrate molecule CRKL undergoes increased phosphorylation. Panel A.** Dual-colour Immunocytofluorescence of CLL lymphocytes probed for F-actin (red) and ABL1 (green)(representative images of CLL lymphocytes with morphological features typical of amoeboid motility). F-actin is particularly enriched within extended projections at the anterior pole of the cell (left panel). ABL1 protein (right panel) shows a fraction with typical nuclear localisation, but an significant additional cytoplasmic fraction (right panel) is co-localised with F-actin (co-incident pixels are shown as yellow in central combined image). This is further highlighted by pixel colocalisation studies, for the image shown, co-localised pixels are indicated in white (ImageJ/FIJI software Colocalisation plugin). **Panel B**. Phosphorylated CRKL protein is increased within cultured cells (mean fluorescence [MF] indicated with each panel). (i) CLL B-cells isolated directly from circulation express low levels of 207P-CRKL. (ii) Cells from culture show an increased 207P-CRKL expression (solid histogram) compared with cells from blood (open histogram) (iii) The large irregular cells (high side scatter fraction) from culture have highest 207P-CRKL expression. **Panel C.** 207P-CRKL phosphorylation does not vary significantly between cases of CLL with IgVH mutation (M-CLL) or without IgVH mutation (UM-CLL); alpha tubulin is shown as a loading control. **Panel D**. Imatinib mesylate inhibits 207P-CRKL-phosphorylation in a dose dependent manner (samples treated with CXCL12 or imatinib at indicated concentration for 30 minutes, 207P-CRKL immunoblot imatinib doses as indicated, alpha tubulin is shown as a loading control).

**Figure 7:**
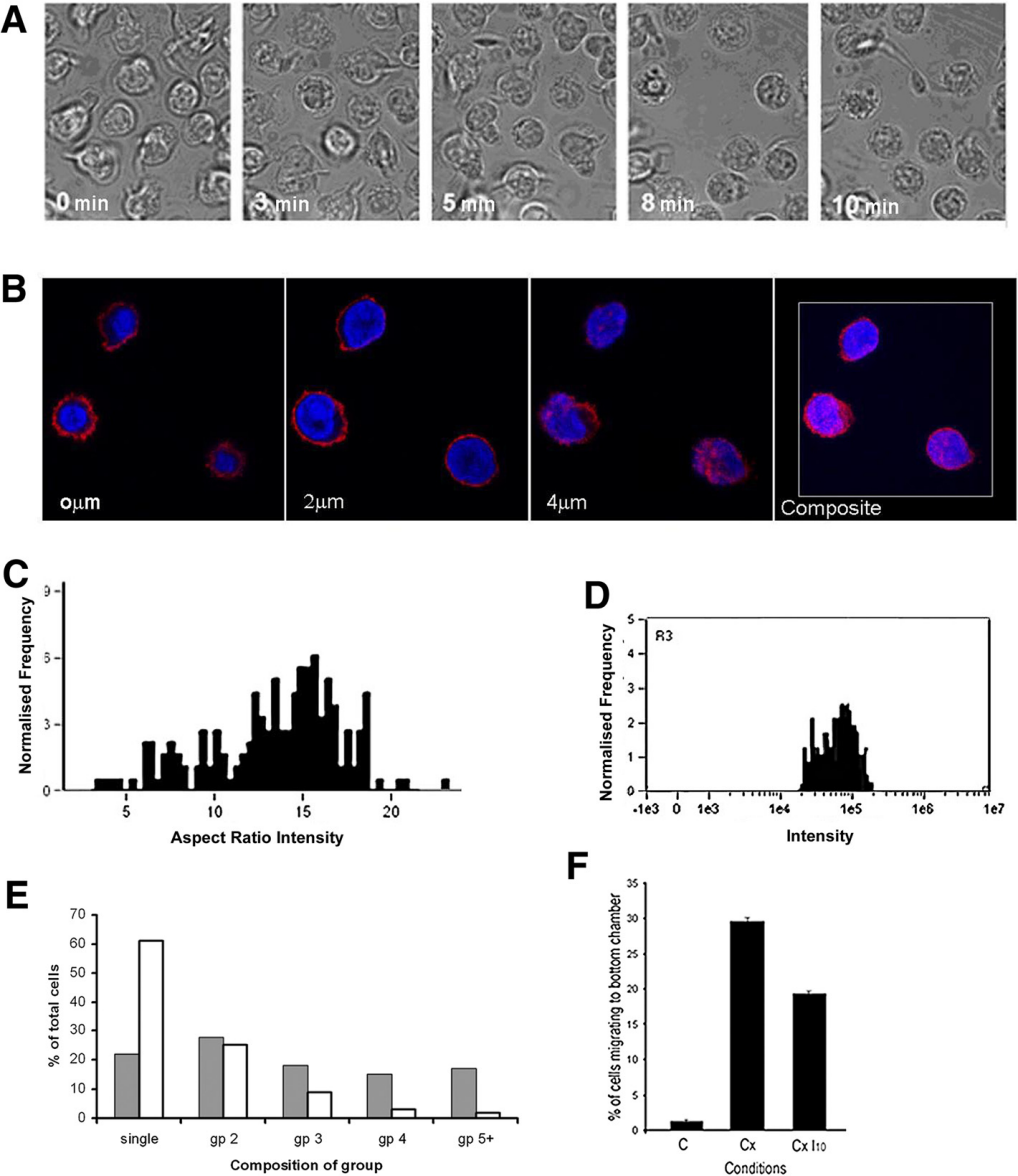
**Inhibition of ABL1 activity using imatinib specifically reverses amoeboid motility and filopodial interaction. Panel A.** Time-interval images of cultured CLL lymphocytes following treatment with imatinib (confocal microscopic images at times indicated) showing the rapid transition from irregular shape with extended projections to a largely round shape with numerous surface microvilli. **Panel B**. Confocal microscopic images of F-actin within imatinib-treated CLL lymphocyte (TR phalloidin probe). The first 3 panels show Z planes through the cell illustrating the narrow F-actin rim and surface projections consistent with the microvilli. The right hand panel is a composite flattened image of all Z-planes. **Panel C**. Shape analysis and F-actin-content of CLL following exposure to imatinib, showing predominantly round cell shapes (a skewed peak of high aspect ratio intensity). **Panel D**. F-actin flow cytometry shows a single peak of F-actin equivalent to the highest expressing population of control cells (NBD-phallacidin probe). **Panel E**. Cell groups of cultured CLL lymphocytes in the absence (solid bars) or presence (open bars) of imatinib. The presence of imatinib causes cells to form fewer and smaller homotypic groups. **Panel F**. Chemotaxis toward CXCL12 is reduced but not prevented by imatinib (filter migration assessed using transwell plate assay). Imatinib-treatment causes a small reduction in the observed migratory fraction, however their migration is not prevented despite using an imatinib dose that prevents amoeboid migration and prevents 207P-CRKL formation.

ABL1 has a substantial role in controlling the F-actin organisation of cells from the immune system, and is recognised to support amoeboid motility and filopodia formation [23]. In CLL the neoplastic cells express high levels of ABL1, which supports their survival *in vitro*[14]. We therefore studied ABL1 localisation and activation during the culture-induced shape change of CLL lymphocytes. Using immunocytofluorescence we showed that a cytoplasmic fraction of ABL1 protein became co-distributed with F-actin within the extended projections of the cell during amoeboid motility (Figure 6A). At the same time, the ABL-substrate molecule CRKL became phosphorylated on the ABL target residue tyrosine-207 (P-CRKL); this phosphorylation was detected particularly within the irregular cells that had high F-actin content (Figure 6B). P-CRKL phosphorylation was not dependent on IgVH mutation status (Figure 6C), and the ABL-inhibitor imatinib mesylate (imatinib) reduced the phosphorylation of CRKL in a dose dependent manner (Figure 6D), and rapidly reversed the culture-induced shape-change of CLL cells: Time-lapse photomicroscopy showed that the imatinib-treated cells retracted their large F-actin-rich processes and reverted to a globular cell-shape with reappearance surface villi, with established effect within 10 minutes (Figure 7A). This morphological change was accompanied by a marked reduction in spontaneous amoeboid migration. Using confocal microscopy we confirmed that imatinib treated cells had a globular appearance, but actin-polymerisation was not prevented, and instead was rearranged, being present in a narrow cortical ring and within frequent F-actin rich villus projections (Figure 7B). Cell shape analysis of cultured cells confirmed a relatively uniform population of cells that had shape parameters that resembled those of cells fixed directly within peripheral blood (Figure 7C), F-actin within the imatinib-treated cells remained highly expressed consistent with its reorganisation into numerous surface microvilli (Figure 7D). At the same time, ABL protein was redistributed within the cells into a non-polar distribution that was separate from the microvillus projections. Imatinib-treated cells formed fewer cell groups (Figure 7E). By contrast however, ABL activity did not appear to play a controlling role in the shape-change and motility induced by chemokine: Although the magnitude of CXCL12-induced cell shape-change was changed in the presence of imatinib, significant cell-elongation was present when compared with imatinib-treated cells not exposed to chemokine, and the proportion of cells that showed F-actin polarisation was identical for chemokine-treated CLL lymphocytes irrespective of the presence of imatinib (66% vs. 62% p = ns Chi Squared test). Similarly, although chemotaxis assays demonstrated that imatinib was able to reduce CXCL12-induced chemotaxis, using doses that abolished 207P-phosphorylation of CRKL did not prevent chemotactic response to the chemokine (Figure 7F). These latter findings are consistent with a separate signal basis for culture-induced and chemokine-dependent cytoskeletal reorganisation, with the former being ABL1 dependent while chemokine effects are at least partly independent of ABL1.

## Discussion

*In vivo*, CLL lymphocytes adapt to the tissue environment, undergoing functional and phenotypic changes that support their migratory and interactive behaviour [8,11,24]. It is probable that similar principles underlie the response of CLL lymphocytes to the *ex vivo* tissue culture environment, and culture systems have successfully been used to model those responses [10]. We were interested particularly in the cytoskeletal changes of the cells within tissues. In the present study we have shown that when fixed directly in whole blood the neoplastic B-lymphocytes of CLL have a uniform globular cell-shape with numerous cell-surface microvilli resembling the features of resting lymphocytes [25]. However, soon after entering high-density culture the cells undergo a rearrangement of their F-actin cytoskeleton that promotes their dynamic motility and homotypic interactions. Using sections of CLL-involved tissues we observed that similar morphological appearances typify CLL lymphocytes within tissues *in vivo*. While, shape change occurring within the tissue environment *in vivo* is likely also to reflect the effects of heterotypic adhesion and chemokine stimulus, the presence of cellular projections extended between CLL cells in tissues was demonstrated. We postulated therefore that the behaviour we observed *in vitro* may in part reflect similar adaptive functional responses occurring *in vivo*.

The mononuclear cell high-density preparations of CLL lymphocytes employed in this study have previously been reported to replicate features of the *in vivo* tissue-environment, and share characteristics with other culture systems used in CLL [10]. In the present study, we observed that a series of dynamic changes affecting cytoskeleton and cell behaviour developed as cells became established in culture. Detailed analysis showed that these morphological forms in fact represented a process of dynamic amoeboid shape-change and “random walk” motility, during which individual cells underwent periodic transition between two morphological forms, a globular cell population and an irregular polarised form in which F-actin polymerisation was increased: the two of apparently different forms in fact representing a single population in dynamic equilibrium. Additionally, during this behaviour the cells extended “exploratory” filopodial processes that mediated specific homotypic contact and which supported the formation of cell-groups. These homotypic cell groups were transient, but comprised cells that had extensive filopodial interactions accompanied by distinctive accumulation of F-actin and integrin receptors at points of contact. When Rho kinase was inhibited to prevent cytoplasmic process retraction [21], extensive interacting networks of extended filopodia were seen to connect the cells, consistent with the involvement of Rho kinase in maintaining the observed structures.

Chemokine induced motility by CLL lymphocytes has been reported and characterised in detail by others, and has been shown to be important during the formation of heterotypic cell interactions, particularly those between CLL-lymphocytes and NLCs [26]. The changes that we observed however were induced at early culture points prior to NLC formation or demonstrable chemokine effect. Furthermore, the morphological and functional characteristics of the culture-associated movement differed from those induced by cytokine. The different cell forms and behavioural responses we have reported are recognised in other lymphocyte types, where they are reported to have a different signal basis and specific functional significance. Elongated-type motility is a directed response that is characteristically induced by chemokine and integrin adhesion has been linked to Rac1-mediated signalling processes associated with directed movement in two dimensions. By contrast, amoeboid motility is a Rho/CDC42 controlled form of cell movement characteristically occurring within three-dimension lattices (reviewed in [27]), and frequently underpins an “exploratory phase” of transient homotypic interactions that accompany early cell activation in tissues. This pattern of movement has been described for normal T lymphocytes and B lymphocytes both *in vivo* and *in vitro*[12,28], and in T lymphocytes the brief homotypic contacts are recognised to mediate cell activation as a prelude to antigen-mediated cell events [29].

Modifying the motility and interactions between CLL lymphocytes and the supportive microenvironment is an attractive therapeutic option in CLL. We therefore explored the signalling basis of the behaviour we observed. In this regard, ABL1 is highly expressed and active in CLL lymphocytes. ABL family kinases bind to F-actin, and are increasingly recognised to play a central role in the control of the F-actin cytoskeletal structures [13,30]. ABL-mediated intracellular signalling affects cell migration in many cell types [13], and specifically mediates interactions of T cells within the immune environment [23]. In the present study the neoplastic cells of CLL were confirmed to express ABL1 as described [14], and the cytoplasmic fraction of ABL1 protein was shown to become co-localised with F-actin within the large projections of motile cells. Inhibition of ABL1 using imatinib caused a rapid dose-dependent inhibition of CRKL-phosphorylation. In parallel with the inhibition of CRKL-phosphorylation the neoplastic cells became globular, with retraction of their extended cytoplasmic projections and a reappearance of their surface microvilli, inducing appearances similar to those of cells fixed in whole blood. Imatinib treated CLL lymphocytes were prevented from undergoing spontaneous motility and filopodial contact between the CLL cells was greatly reduced. Consistent with this a separate signal basis for elongated-type motility, imatinib had significantly less effect on chemokine-induced behaviour, such that cell polarisation and elongation continued to occur even in the presence of the drug and significant chemotaxis was observed at imatinib concentrations that significantly inhibited CRKL phosphorylation.

Several threads link our own findings to pathways and behaviours that have recognised importance to CLL biology and to CLL cytoskeletal-function. ABL-family kinases interact directly with the F-actin control complex that includes WASP/WAVE2 proteins [31], and through these proteins to activation of actin-nucleation proteins (ARP1 and ARP2) that mediate cytoskeletal reorganisation. The microvillus F-actin structure of lymphocytes in circulation is independent of WASP/WAVE2 and therefore would not be affected by ABL1 inhibition, and may be regarded as a “default” organisation of lymphocyte cytoskeleton [25]. However, ABL1 activation is central to the formation of structures that include lamellipodia and filopodia [32,33]. The reversion of CLL cytoskeleton to microvillus structure following imatinib-treatment of CLL lymphocytes therefore, is entirely consistent with an importance for ABL1-dependent signalling in the cells. In this regard, ABL-family kinases also interact with several other pathways that have emerging importance in CLL biology. The LYN kinase substrate protein HS1 that has a major role in cytoskeletal activation in CLL is recognised to act together with ABL1/CRKL and ZAP70 to induce cytoskeletal activation in lymphoid cells [23]. This study reinforces the complexity of the motile behaviour and cellular interactions made by CLL lymphocytes identifying separate motile and interactive behaviours linked either to spontaneous movement or to chemokine. ABL1 signals may therefore be considered as one part of a system of signal processes and cellular interactions that act together to drive the pathophysiology of the disorder [34]–[36].

Interest in CLL motility and migration has recently increased following the recognition that the therapeutic effect of signal inhibitors including BTK inhibitors is accompanied by a compartment shift of cells between tissue and blood, suggesting that effects on cell interactions or migration may contribute to the therapeutic benefit of these agents [16]. However, despite ABL1 inhibition having significant activity against CLL *in vitro* affecting a range of cell behaviours [14,37,38], single agent inhibition of ABL1 by imatinib or dasatinib has not emerged as having major clinical activity *in vivo*[39] The present study however emphasises however, that the interaction between CLL cells and their tissue microenvironment is complex, and that ABL1 may form part of a coordinated behaviour pattern that together with other pathways promotes the migration and tissue interactions of the neoplastic cells. The findings of *in vitro* studies may not be directly applicable the more complex microenvironment of the cells *in vivo*, and the functional significance of our findings to the pathobiology of CLL remains to be established in patients. However, awareness of how these different pathways contribute to the microenvironment interactions in CLL is therefore important, and should be considered as coordinated strategies designed to disrupt environment interactions are developed.

## Conclusions

The present study demonstrates for the first time that activation of ABL1-linked signal pathways is an early response by CLL lymphocytes within the cell culture environment, that is associated with amoeboid-pattern migration and with frequent brief homotypic interactions between the neoplastic cells. This behaviour is distinct from the responses of CLL lymphocytes to chemokine, and from those which occur during heterotypic interactions. We suggest that ABL1 mediated migration and interactions may be an early adaptive response by CLL cells leaving the circulation, and may resemble similar behaviour by T-lymphocytes where such interactions promotes their activation, and enhance their subsequent response to antigen or other stimuli. Awareness of the different pathways that together coordinate the motility and interaction in CLL within the tissue microenvironment is highly relevant to the development of new treatment strategies, and targeting ABL1 may have a role as part of such approaches.

## Methods

### Cells and preparation

Detailed evaluation employed samples from 8 patients, confirmatory study of morphology and of further biochemical aspects used samples from 10 additional patients. Patient samples were collected with informed consent with approval of the local Research Ethics Committee (reference 10/H1017/73). All samples had typical clinical presentation and immunophenotype with no adverse cytogenetic features, and comprised >95% CLL B-lymphocytes as determined by flow cytometry. Specific samples differing in the presence or absence of somatic mutation of IgVH genes were kindly provided from the Leukaemia Lymphoma Research -Cell Bank Facility at the Royal Liverpool University Hospital. Tissues from diagnostic use were employed to examine cell interactions within tissue sections. Mononuclear cells were isolated by Ficoll-Paque gradient centrifugation (GE Healthcare, Buckinghamshire, UK) and were used freshly or were cryopreserved until use in 90% fetal bovine serum, 10% DMSO cells.

### Culture/culture assays

Cells were maintained in culture at 1 × 10^7^/ml in 24 well plates unless otherwise indicated using RPMI medium with L-glutamine (Invitrogen, Paisley, UK) and 10% FBS supplemented with penicillin and streptomycin. Where adherent cells were required, the cells were washed and cells were induced to adhere directly to fibronectin (FN)-coated glass coverslips (40 μg/ml overnight) within the 24 well plates in serum-free conditions for the duration of the experiment. Chemotaxis experiments employed filter migration plates with pore size 5 μm (Transwell Plates, Corning Life Sciences, Amsterdam, The Netherlands) initial cell density in the upper chamber was 5 × 10^6^/ml. Cells migrating into the bottom plate were counted after the indicated time. For all culture assays, reagents and drugs were added directly to culture medium at the indicated final concentrations.

### Antibodies and biochemicals

General biochemicals and reagents were obtained from Sigma Aldridge (Sigma Aldridge, Poole, UK) unless otherwise specified. Fluorochrome-conjugated antibodies recognising the following antigens were employed: CD19, CD5, CD23, CD14, CD3, CD4 all from (BD Pharmingen, Oxford, UK). Unconjugated antibodies recognised: CD29 (clone P5D2 Abcam, Cambridge, UK), CRKL (ab32018 Abcam), P-CRKL (Y207) ( #3181 Cell Signaling Technology, New England Biolabs, Hitchin, UK), ABL (ABL clone 8E9 Abcam), ARG (181B11 Abcam). Secondary antibodies used for immunofluorescence were (Goat anti-mouse FITC Ab5999 and goat anti-mouse tr ab6003 Abcam). Phalloidin probes for F-actin were nitrobenzoxadiazole (NBD)-pallacidin (Invitrogen) and Texas Red phalloidin (Invitrogen). Other agents were CXCL12 (Peprotech EC Ltd, London, UK); Y27632 (Tocris Bioscience, Bristol, UK); and imatinib (LKT Laboratories, Alexis Corporation, Lausen, Switzerland). Annexin V FITC Staining kit (BD Pharmingen) was used with manufacturers protocols to determine apoptotic cell death. Immunoperoxidase staining used standard reagents and protocols (DAKO UK Ltd, Ely, UK).

### Slide imaging and slide immunofluorescence

Live cell observation employed enclosed, temperature and CO2 controlled environment. Cells were taken from high density culture and added to glass-bottomed 24 well culture plates for the period of the experiment only. Cell density was reduced to allow optimal viewing within optimal areas. Established shape transition was not reversed during the culture period allowing cells to be observed in detail. Image capture as single image or time-lapse series employed a dLeica DMIRE2 using × 63 oil lens (Leica Microsystems, Milton Keynes, UK) image capture employed Image-Proplus (Media Cybernetics Inc., Bethesda, MD) with bright field or interference settings, image series were analysed using ImageJ (public domain open source software). General viewing of fixed-stained cells used bright field or immunofluorescence using an Nikon Eclipse 80i microscope with camera Hamamatsu C4742-95 camera or Nikon DSFi1 equipped with NIS-Elements BR 2.30 software/image analysis modules (supplied by Nikon, Nikon Instruments Europe, Amstelveen, The Netherlands). Plane images of brightfield sections used a Zeiss Axio Imager M.1 with AxioCam and Plan-apochromatic 63× 1.4 oil lens and AxioVision software 4.8.1 (Karl Zeiss Ltd, Herts, UK). Confocal microscopy employed a Zeiss LSM510META Confocal microscope with × 100 (1.4NA0) lens (Zeiss LSM 501META/LSM software, Karl Zeiss Ltd). Cells from adherence culture were fixed for 10 min in 4% w/v paraformaldehyde then washed in PBS, coverslips were then extracted for individual staining. Image analysis samples were stained using Rose Bengal 1% in PBS. Cell shape measures used approaches previously described [40], and were made using NIS-Elements BR 2.30 (Nikon). Optimal shape measures to discriminate shape of adherent cells were tested and cell area and cell elongation provided best discrimination (and was comparable to aspect ratio intensity measured by flow cytometry described below). Measures were exported for further analysis (SPSS/Microsoft Excel). Samples for immunostaining or F-actin probes were permeablised in 0.2% v/v Triton-X for 5 min before blocking in PBS/1%BSA (w/v). Primary antibodies were diluted in buffer and titrated to optimal concentration and used alone or in combination as indicated followed by appropriate secondary antibodies. All slides were mounted in Prolong Gold Antifade reagent with DAPI (4′,6-diamidino-2-phenylindole)(Invitrogen) for examination. Additional image processing employed ImageJ/JAVA software (ImageJ FIJI v1.48 k NIH, US) using Colocalization Plugin for pixel co-localisation analysis.

### Flow cytometry

Standard flow cytometery used a FACSCanto II with BD FACSDiva Software using standard protocols and reagents (BD Biosciences). Morphological analysis by flow cytometry employed the ImageStream Imaging Flow Cytometer (Amnis, Seattle, WA) using simultaneous imaging facility and analysis by IDEAS v4 software (Amnis). To allow determination of shape change live cells were simultaneously fixed and stained using NBD phallacidin. Different shape parameters were tested for their capability to discriminate cell populations. Aspect ratio intensity (a measure that combines weighted actin intensity distribution and elongated cell shape) provided the most effective discrimination, between cell populations. Parameters were exported for detailed analysis using spreadsheet software (Microsoft Excel).

### Biochemical analysis

Cell lysates were prepared from washed cultured cells using RIPA buffer and inhibitors (Halt protease and phosphatase Thermo scientific/Pierce, Cramlington, UK). Samples (5 μg) were separated using SDS-PAGE and 10% gels. The separated proteins were transferred onto polyvinlidene fluoride (PDVF) membrane (GE Healthcare) then washed with PBS-Tween (1xPBS with 1% Tween 20) and blocked (5% w/v non-fat dry milk). Washed membranes were incubated with primary antibody as indicated. HRP-conjugated secondary antibody (GE Healthcare) was detected using Enhanced Chemiluminescence (GE Healthcare).

### Statistical analysis

Statistical testing was performed as indicated in individual experiments using SPSS software (IBM-SPSS, Portsmouth, UK).

## Abbreviations

CLL: Chronic lymphocytic leukaemia; Imatinib: Imatinib mesylate; F-actin: Filamentous actin; FN: Fibronectin; NBD: Nitrobenzoxadiazole; DAPI: 4′,6-diamidino-2-phenylindole; NLC: Nurse-like cell.

## Competing interests

The authors declare that they have no competing interests.

## Authors’ contributions

CVH designed and carried out and interpreted experiments, and helped draft the manuscript. SN and SJ performed experiments, JA performed and interpreted experiments, KRU performed and interpreted experiments and critically evaluated the manuscript. JB designed the work, interpreted data and wrote the manuscript. All authors read and approved the final manuscript.
